# Sociodemographic factors and pregnancy outcomes associated with prepregnancy obesity: effect modification of parity in the nationwide Epifane birth-cohort

**DOI:** 10.1186/s12884-017-1456-8

**Published:** 2017-08-25

**Authors:** Julie Boudet-Berquier, Benoit Salanave, Jean-Claude Desenclos, Katia Castetbon

**Affiliations:** 1 Nutritional Surveillance and Epidemiology Team (ESEN), French Public Health Agency, Paris-13 University, Centre de Recherche en Epidémiologie et Statistiques, COMUE Sorbonne Paris Cité, SMBH Building, 1st floor, door 136, 74 rue Marcel Cachin, 93017 Bobigny Cedex, France; 2 French Public Health Agency (Agence nationale de Santé publique), Saint Maurice, France; 3 Centre de Recherche « Epidémiologie, Biostatistique et Recherche clinique », School of Public Health, Université libre de Bruxelles (ULB), Brussels, Belgium

**Keywords:** Adverse pregnancy outcomes, Maternal obesity, National birth cohort, Social inequalities

## Abstract

**Background:**

In light of the adverse outcomes for mothers and offspring related to maternal obesity, identification of subgroups of women at risk of prepregnancy obesity and its related-adverse issues is crucial for optimizing antenatal care. We aimed to identify sociodemographic factors and maternal and neonatal outcomes associated with prepregnancy obesity, and we tested the effect modification of parity on these associations.

**Methods:**

In 2012, 3368 mothers who had delivered in 136 randomly selected maternity wards were included just after birth in the French birth cohort, Epifane. Maternal height and weight before and at the last month of pregnancy were self-reported. Maternal and neonatal outcomes were collected in medical records. Prepregnancy Body Mass Index (pBMI) was classified into underweight (<18.5), normal (18.5-24.9), overweight (25.0-29.9) and obesity (≥30.0). Since we found statistically significant interactions with parity, the multinomial logistic regression model estimating associations of pBMI class with sociodemographic characteristics and pregnancy outcomes was stratified on parity (1335 primiparous and 1814 multiparous).

**Results:**

Before pregnancy, 7.6% of women were underweight, 64.2% were of normal weight, 18.0% were overweight and 10.2% were obese. Among the primiparous, maternal age of 25-29 years (OR = 2.09 [1.13-3.87]; vs. 30-34 years), high school level (OR = 2.22 [1.33-3.73]; vs. university level), gestational diabetes (OR = 2.80 [1.56-5.01]) and hypertensive complications (OR = 3.80 [1.83-7.89]) were independently associated with prepregnancy obesity. Among the multiparous, primary (OR = 6.30 [2.40-16.57]), junior high (OR = 2.89 [1.81-4.64]) and high school (OR = 1.86 [1.18-2.93]) education levels (vs. university level), no attendance at antenatal classes (OR = 1.77 [1.16-2.72]), excess gestational weight gain (OR = 1.82 [1.20-2.76]), gestational diabetes (OR =5.16 [3.15-8.46]), hypertensive complications (OR = 8.13 [3.97-16.64]), caesarean delivery (OR = 1.80 [1.18-2.77]) and infant birth weight ≥ 4 kg (OR = 1.70 [1.03-2.80]; vs. birth weight between 2.5 kg and 4 kg) were independently associated with prepregnancy obesity.

**Conclusion:**

Obesity before pregnancy is associated with a set of sociodemographic characteristics and adverse pregnancy outcomes that differ across parity groups. Such findings are useful for targeted health policies aimed at attaining healthy prepregnancy weight and organizing perinatal care.

**Electronic supplementary material:**

The online version of this article (doi:10.1186/s12884-017-1456-8) contains supplementary material, which is available to authorized users.

## Background

Since women entering pregnancy with obesity may face adverse health issues affecting themselves and their offspring [1], maternal obesity is now a crucial public health problem worldwide [2]. Studies have provided strong evidence of the association of maternal obesity with risk of gestational diabetes mellitus (GDM), pre-eclampsia, caesarean delivery and large-for-gestational-age newborns [3–6]. Moreover, pre-existing type 2 diabetes [7] may be involved in higher risk of congenital malformations associated with maternal obesity [8]. Preterm birth and small- and large-for-gestational-age birth might also partially mediate the association observed between maternal obesity and higher risk of stillbirth and infant death [9]. In addition, recent observational studies suggested the involvement of maternal obesity in the risk, for the offspring, to develop obesity during adulthood independently of their adult lifestyle factors [10], and to suffer from premature mortality related to cardiovascular events [11].

In view of these adverse health issues, identification of sociodemographic characteristics associated with maternal obesity is useful for implementing targeted preventive actions and improving their efficacy. However, the relationship between sociodemographic factors and prepregnancy body mass index (pBMI) appears inconsistent across studies. Poor socioeconomic conditions (living in a low income household [5] or in a deprived area [12, 13]) have consistently been found to be risk factors in maternal obesity. In contrast, inconsistent results have been found in the association of maternal obesity with age at giving birth [12, 14] and educational level [5, 12].

A number of studies have indicated that parity was a risk factor in maternal obesity [5, 12, 14]. Several authors have investigated underlying mechanisms involved in the relationship between childbearing and development of obesity, including post-partum weight retention [15–17]. Excessive gestational weight gain (GWG) and a short time lapse between pregnancies have been shown to be independently associated with postpartum weight retention and further maternal obesity [16]. Weight gained during pregnancy and after birth might depended not only on prepregnancy body weight and hormonal changes, but also on modifications in lifestyle due to child-rearing and socioeconomic factors [18]. Such considerations suggest that the association of sociodemographic characteristics with the risk of maternal obesity may differ by parity. In addition, the pattern of adverse health outcomes associated with prepregnancy weight have been shown to differ between primiparous and multiparous women [19]. A better understanding of the role of parity in the relationship between maternal obesity and adverse pregnancy issues would therefore optimize perinatal care.

However, studies investigating the effect modification of parity in the association between maternal obesity and sociodemographic factors and maternal and neonatal outcomes are scarce. Using data from the French nation-wide birth cohort Epifane, performed in 2012, our objectives were: (1) to assess sociodemographic characteristics and maternal, fetal and neonatal outcomes associated with maternal obesity; and (2) to investigate the effect modification of parity by testing the interaction between parity and sociodemographic and medical factors. Our hypotheses were that: (1) in France, in 2012, sociodemographic characteristics and maternal and neonatal outcomes in obese mothers differ from those of normal-weight women; (2) such associations vary between primiparous and multiparous women.

## Methods

### Study design

We have previously described the study methods [20]. Briefly, Epifane was a nation-wide birth cohort based on two-stage random sampling. First, 136 maternity wards in mainland France were randomly selected proportionally to the yearly number of deliveries and stratified on the private/public status, equipment level of the maternity hospital and five geographic areas. Then, after verifying eligibility criteria, 25 mother-infant dyads per maternity ward were included one or two days after delivery. Eligibility criteria for mothers were the following: age over 18, not institutionalized, able to speak, read or write French or to get help from someone who did. The newborn had to be born at 33 amenorrhea weeks (AW) or later, without severe pathology requiring hospitalization, and had not been transferred to a unit other than the maternity ward in the days following birth. A total of 3368 dyads from 136 maternity wards were included between January and April 2012. Mothers and midwives filled out a questionnaire at the maternity ward, and each mother-infant dyad was then followed up during the child’s first year. Thus, mothers were contacted by phone at 1, 4, 8 and 12 months post-partum. The Epifane cohort project was approved by the Committee for Data Processing in Health Research (CCTIRS, registration n°11.335) and the French Data Protection Authority (CNIL, authorization n°911,299).

### Prepregnancy body mass index (pBMI)

Our outcome of interest was the pBMI. It was calculated using self-reported height and weight before pregnancy: weight before pregnancy (kg)/height (m^2^). The pBMI was grouped into 4 classes according to the WHO classification [21]: underweight (<18.5), normal weight (18.5-24.9), overweight (25.0-29.9) and obesity (≥30.0).

### Parity status

At 1 month post-partum, mothers indicated the total number of their biological children, comprising the newborn included in the Epifane cohort. Based on this information, we categorized parity as primiparous women (the women for whom the newborn included in the Epifane cohort was the first child) and multiparous women (the women for whom the newborn included in Epifane was at least the second).

### Covariates

We studied the association of the pBMI with a set of different factors, including sociodemographic characteristics, health behaviors, and maternal and neonatal outcomes.

#### Gestational weight gain during pregnancy (GWG) according to IOM recommendations

GWG was defined as the difference between prepregnancy weight and weight in the last month of pregnancy, declared by mothers. The 2009 Institute of Medicine (IOM) recommendations [22] define adequate GWG based on pBMI as follows: between 12.7 and 18.1 kg for women with prepregnancy underweight; 11.3 and 15.9 kg for women of normal weight; 6.8 and 11.3 kg for those who are overweight; 5.0 and 9.7 kg for women with prepregnancy obesity. In accordance with these recommendations, a three-category variable indicated whether the woman met these recommendations: GWG within IOM recommendations, GWG lower than IOM recommendations and GWG higher than IOM recommendations.

#### Demographic and socioeconomic factors

Maternal age, maternal country of birth, marital status (married/not married), education level (primary school, junior high school, high school, university) and occupation (farmer/craftswoman/merchant, manager, intermediate occupation, manual worker, unemployed) were collected using a questionnaire completed by the mothers at the maternity ward. We categorized maternal age using classes already used in national [23] and international [3] studies: 18-24 years, 25-29 years, 30-34 years and ≥35 years.

At each phone interview during the child’s first year, mothers were asked if they had returned to work. If so, they specified the exact date in days and months. We used a categorical variable: return to work before the infant was 4 months old, between 4 and 6 months old, between 6 and 12 months old, or did not return to work 12 months after child’s birth.

#### Health behaviors during pregnancy

Tobacco consumption (did not smoke before and during pregnancy; smoked before but not during pregnancy, smoked before and during pregnancy), any alcohol consumption during pregnancy (yes/no) and attendance at antenatal classes (yes/no) were also self-reported at the maternity ward.

#### Maternal, fetal and neonatal outcomes

Maternity midwives collected information on pregnancy and birth conditions from health records. We selected maternal, fetal and neonatal outcomes which had been found to be associated with maternal obesity in the literature [1, 3–5]. Maternal and fetal conditions included maternal hypertensive complications during pregnancy (including hypertension and/or pre-eclampsia during pregnancy), other complications during pregnancy (premature delivery threat, fetal growth restriction, bleeding, preterm rupture of membranes, etc.), congenital defects and mode of delivery. In addition, diagnosis of GDM was declared by the mother and by the midwife at the maternity ward. In case of inconsistent answers between the mother and the midwife, mothers were considered as suffering from GDM when the midwife reported it; or when the mother reported suffering from GDM and subsequently described the medical care received to manage it. French guidelines concerning GDM were updated in 2010, recommending targeted screening of high-risk women, including women with pBMI higher than 25 kg/m^2^ [24]. The screening process comprises a fasting blood glucose tolerance test during the first trimester of pregnancy and a 75 g Oral Glucose Tolerance Test (OGTT) between 24 and 28 gestational weeks. Diagnostic criteria for GDM include a fasting blood glucose level ≥ 0.92 g/L (5.1 mmol/L), and/or a 1-h-after-OGTT blood glucose level ≥ 1.80 g/L (10.0 mmol/L) and/or a 2-h-after-OGTT blood glucose level ≥ 1.53 g/L (8.5 mmol/L). Based on the Epifane cohort, however, 15.3% of overweight women and 9.8% of obese women were not screened [25]. Neonatal outcomes included gestational age at birth, sex, birth weight and Apgar score 5 min after birth collected in the health record.

### Statistical analyses

In total, 18.6% of questionnaires in our sample (*n* = 628) had missing values for occupation; “missing information” was retained as a full category. To address missing data on sociodemographic characteristics with a rate of missing data above 5% (maternal country of birth, education, and parity), logistic regression models were performed, including, when appropriate, maternal age, marital status, parity, education, occupation and partner’s education. Missing values for tobacco consumption before and during pregnancy (*n* = 12) were imputed using the mode “no smoking before and during pregnancy.” We did not impute missing data concerning pregnancy outcomes (*n* = 59); thus, only dyads with available information concerning pregnancy and delivery outcomes were included in analyses.

Weights were first calculated so as to take into account inclusion probabilities. To provide statistical estimates representative of the source population, marginal calibration was then performed on maternal age, matrimonial status, level of education (as a binary variable, level of education equal or lower than high school or higher high school graduation) and type of pregnancy (multiple or single). Percentages observed in the French National Perinatal Survey 2010 were used as references, since they had been validated against vital statistics [23]. To take into account the random complex sampling design, the stratification variable and final weights were taken into account in all analyses using the “svyset” command, (Stata® V12.1).

We used multinomial logistic regression to identify factors associated with pBMI, normal weight status (pBMI between 18.5 and 24.9) being the reference class. In bivariate analyses, demographic and socioeconomic factors, health behaviors during pregnancy and maternal, fetal and neonatal outcomes were compared by pBMI category using the adjusted Wald test. Variables associated with pBMI with a *p*-value < 0.20 were included in the initial multinomial logistic regression model. The final model included covariates selected after using a manual back stepwise procedure, and significantly associated with pBMI with a *p*-value < 0.05. Nonetheless, a covariate was retained if its removal led to a variation in the odds ratio (OR) above 10%.

In order to assess an effect modification of parity (primiparous/multiparous) with each covariate of the pBMI categories, we performed multinomial logistic regression models, including interaction terms such as “parity * sociodemographic covariate” and “parity * pregnancy or delivery outcome”. Interaction *p*-values in the adjusted model are presented in the Additional file 1: Table S1. Since interactions of parity with maternal age (*p*-value = 0.02), maternal education (*p*-value = 0.08), and tobacco consumption (*p*-value = 0.08) could be considered statistically relevant (*p*-value < 0.10 [26]), in the final model, we stratified the adjusted multinomial on parity. Analyses were performed using Stata (version 12.1). Odds ratios were estimated with a 95% confidence interval.

We ran a set of sensitivity analyses. First, analyses were carried out in a sample of women with available information concerning parity status (*n* = 2888). Secondly, in order to address the issue of possible overadjustment in the association between maternal obesity and caesarean section, multivariate models excluding infant birthweight, hypertensive complications and GDM were estimated. Indeed, these factors themselves have been found to be associated with caesarean section [27].

## Results

### Study sample

**Fig. 1:**
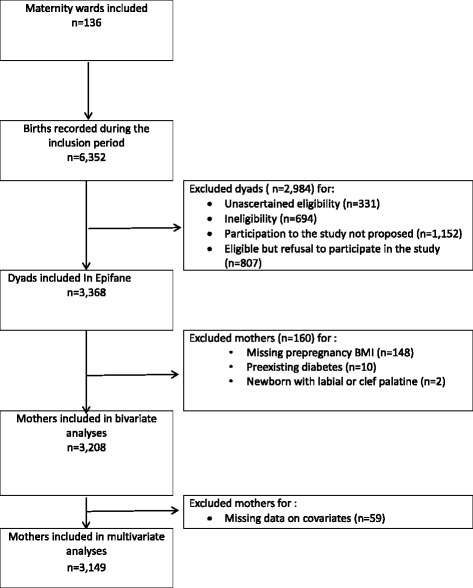
Flow diagram for selection of mothers for the present study (Epifane birth cohort)

Among 3368 mothers included, 3220 completed information on prepregnancy weight and height (Fig. 1). Twelve were excluded from the present analyses because they suffered from pre-existing diabetes (*n* = 10) or their child had cleft lip and/or palate (*n* = 2), leading to 3208 dyads included in bivariate analyses. Further, 3149 mothers were included in multivariate analyses due to missing values for some maternal and neonatal outcomes.

### Subject characteristics

**Fig. 2:**
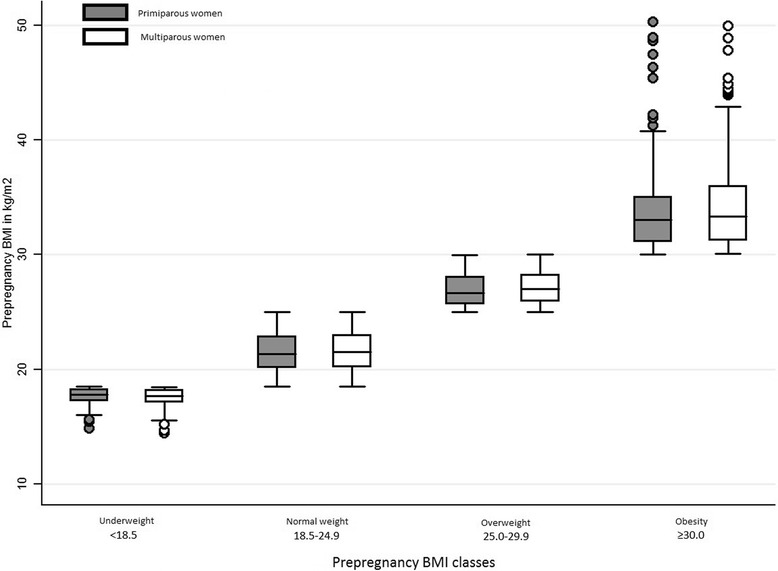
Distribution of prepregnancy BMI according to classes defined by WHO classification, among primiparous and multiparous women (*n* = 3208)

Most mothers were 25-34 years old, born in France, with university level education (Table 1). About half were first-time mothers. Before pregnancy, 7.6% were underweight (*n* = 240), 64.2% of normal weight (*n* = 2067), 18.0% overweight (*n* = 583) and 10.2% had obesity (*n* = 318). Medians of pBMI and interquartiles ranges in each pBMI class defined by the WHO classification are presented for primiparous and for multiparous women in Fig. 2. Thirty-seven percent of women gained more weight during pregnancy than IOM recommendations (Table 2). Slightly less than 80% of women delivered vaginally. Approximately 90% gave birth to newborns weighing between 2.5 kg and 4 kg. Gestational diabetes occurred in 7.7% of women and hypertensive complications in 3.5% (Table 2).

**Table 1:** Maternal sociodemographic characteristics and health behavior, overall and across-prepregnancy body mass index classes (*n* = 3208)

		Prepregnancy BMI				
	All	Underweight	Normal weight	Overweight	Obesity	*p*-value^a^
	*n* = 3208	*n* = 240	*n* = 2067	*n* = 583	*n* = 318	
%						
Maternal age (years) (*n* = 3208)						
18-24	17.0	28.6	15.9	16.2	16.9	<10^−3^
25-29	32.9	34.2	32.1	34.2	34.5	
30-34	31.1	26.6	33.0	30.8	22.9	
≥ 35	19.0	10.7	19.0	18.8	25.7	
Maternal country of birth (*n* = 3208)						
France (mainland and overseas)	83.8	83.3	84.9	82.7	79.1	0.03
Africa	8.7	5.0	7.9	11.8	11.4	
Europa, Asia, America, Oceania	7.5	11.7	7.2	5.5	9.5	
Parity (*n* = 3208)						
1 child	43.3	48.1	45.4	37.3	37.3	<10^−3^
2 children or more	56.7	51.9	54.6	62.7	62.7	
Marital status (*n* = 3208)						
Married	47.7	42.0	47.0	51.7	49.7	0.08
Unmarried	52.3	58.0	53.0	48.3	50.3	
Maternal education (*n* = 3208)						
Primary school	2.4	5.4	1.2	4.0	5.1	<10^−3^
Junior high school	20.7	22.6	18.5	21.8	30.7	
High school	23.6	21.2	22.5	25.7	29.5	
University	53.3	50.8	57.8	48.5	34.7	
Maternal occupation (*n* = 3208)						
Farmer, craftswoman, merchant	2.1	2.2	2.5	0.8	2.4	<10^−3^
Management profession	15.6	13.0	18.5	10.6	8.2	
Intermediate profession	47.1	44.9	46.6	51.4	43.2	
Manual worker	7.3	6.8	7.0	6.4	10.9	
Unemployed	6.8	4.0	5.8	9.8	10.1	
Missing	21.1	29.1	19.5	21.0	25.2	
Time of return to work^b^ (*n* = 3208)						
<= 4 months	34.4	32.5	37.1	29.9	27.5	<10^−3^
]4 months-6 months]	12.5	14.4	13.2	10.0	11.3	
]6 months-12 months]	24.4	26.9	23.9	27.1	20.9	
Did not go back at 12 months	28.7	26.2	25.9	32.9	40.3	
Smoking before/during pregnancy (*n* = 3208)						
No smoking before or during	67.6	62.0	66.8	70.4	71.3	0.07
Smoking before but not during	15.7	14.8	16.7	13.5	14.1	
Smoking before and during	16.7	23.2	16.5	16.1	14.7	
Alcohol during pregnancy (*n* = 3198)						
No consumption	93.9	94.9	93.6	94.1	93.9	0.87
Consumption	6.1	5.1	6.4	5.9	6.1	
Antenatal classes (*n* = 3205)						
Attended	53.2	51.2	56.8	48.8	39.0	<10^−3^
Not attended	46.8	48.8	43.2	51.2	61.0	

^a^Adjusted Wald test *P*-value for comparisons across pBMI classes

^b^Defined as time when the mother returned to work after birth

**Table 2:** Maternal and neonatal outcomes, overall and across-prepregnancy body mass index (*n* = 3208)

		Prepregnancy BMI				
	All	Underweight	Normal weight	Overweight	Obesity	*p*-value^a^
	*n* = 3208	*n* = 240	*n* = 2067	*n* = 583	*n* = 318	
%						
Type of pregnancy (*n* = 3208)						
Multiple	1.3	0.9	1.3	1.5	1.6	0.86
Single	98.7	99.1	98.7	98.5	98.4	
Mean of GWG (in kg) (*n* = 3162)						
	13.2	14.5	13.9	12.6	8.7	<10^−3^
Gestational weight gain (*n* = 3162)^b^						
Within IOM	35.5	48.8	36.5	29.5	29.0	<10^−3^
Below IOM	27.5	35.5	31.5	12.1	23.5	
Above IOM	37.0	15.7	32.0	58.3	47.5	
Gestational diabetes mellitus (*n* = 3204)						
No	92.3	98.6	94.4	88.8	80.7	<10^−3^
Yes	7.7	1.4	5.6	11.2	19.3	
Hypertensive complications^c^ (*n* = 3204)						
No	96.5	98.4	97.6	96.8	87.4	<10^−3^
Yes	3.5	1.6	2.4	3.2	12.6	
Delivery mode (*n* = 3200)						
Vaginal	81.4	89.6	82.9	77.5	73.2	<10^−3^
Cesarean	18.6	10.4	17.1	22.5	26.8	
Gestational age at birth (*n* = 3183)						
≥ 37 amenorrhea weeks	96.4	94.3	96.5	96.8	96.3	0.50
33-36 amenorrhea weeks	3.6	5.7	3.5	3.2	3.7	
Infant’s sex (*n* = 3200)						
Male	49.4	48.6	49.6	49.6	47.8	0.95
Female	50.6	51.4	50.4	50.4	52.2	
Infant’s birth weight (*n* = 3202)						
[2.5 kg-4 kg[	89.0	85.7	89.8	89.1	86.4	<10^−3^
≥4 kg	7.4	3.8	6.8	8.8	11.1	
<2.5 kg	3.6	10.5	3.4	2.2	2.6	
Apgar score at 5 min (*n* = 3181)						
10	94.7	96.5	95.2	93.2	93.6	0.40
8-9	4.6	3.1	4.4	5.9	4.8	
≤7	0.7	0.3	0.4	1.0	1.6	

^a^Adjusted Wald test *P* value for comparisons across pBMI classes

^b^Gestational weight gain in agreement with recommendations defined in 2009 by the IOM

^c^Hypertensive complications including hypertension and/or preeclampsia during pregnancy

Except for alcohol consumption, all demographic, socioeconomic and health behavior characteristics were associated with pBMI categories in bivariate analyses, with a *p*-value under 0.20 (Table 1). In addition, GWG, GDM, hypertensive complications, delivery mode and birth weight were associated with pBMI category with a *p*-value under 0.20 (Table 2). In preliminary multivariate analyses, maternal age and birthplace, parity, education level, occupation, tobacco status, attendance at antenatal classes, GWG, GDM, hypertensive complications, mode of delivery and birth weight were significantly associated (*p*-value < 0.05) with pBMI class (Data not shown). Since we found a statistically significant interaction (*p*-value < 0.10) between parity and maternal age (*p*-value = 0.02), parity and maternal education (*p*-value = 0.08), and parity and tobacco consumption (*p*-value = 0.08) we present here only the adjusted model stratified on parity (Tables 3 and 4).

**Table 3:** Association of sociodemographic factors with prepregnancy BMI category (multinomial regression model stratified on parity) (*n* = 3149)

	Primiparous (*n* = 1335)						Multiparous (*n* = 1814)					
	Underweight		Overweight		Obesity		Underweight		Overweight		Obesity	
	*n* = 107		*n* = 214		*n* = 114		*n* = 130		*n* = 357		*n* = 197	
	OR	[95% CI]	OR	[95% CI]	OR	[95% CI]	OR	[95% CI]	OR	[95% CI]	OR	[95% CI]
Maternal age (years)												
18-24	2.07	[0.97-4.39]	0.80	[0.48-1.32]	1.27	[0.57-2.85]	3.03	[1.57-5.87]	0.73	[0.39-1.37]	0.98	[0.49-1.97]
25-29	1.55	[0.85-2.82]	0.69	[0.46-1.04]	2.09	[1.13-3.87]	1.28	[0.80-2.05]	1.39	[1.01-1.93]	1.11	[0.72-1.72]
30-34	1		1		1		1		1		1	
≥ 35	0.84	[0.30-2.35]	0.70	[0.37-1.33]	2.01	[0.94-4.28]	0.74	[0.42-1.32]	1.04	[0.73-1.48]	1.45	[0.93-2.25]
Maternal country of birth												
France (mainland and overseas)	1		1		1		1		1		1	
Maghreb and Sub-Saharan Africa	1.09	[0.34-3.49]	0.99	[0.37-2.67]	0.81	[0.25-2.61]	0.54	[0.17-1.67]	1.31	[0.78-2.20]	0.77	[0.43-1.38]
Europe, Asia, America, Oceania	3.16	[1.52-6.54]	0.75	[0.34-1.67]	0.78	[0.22-2.79]	1.21	[0.50-2.89]	0.49	[0.22-1.09]	1.64	[0.79-3.41]
Maternal education												
Primary school	3.73	[1.10-12.68]	1.77	[0.53-5.91]	0.69	[0.09-5.38]	3.21	[1.01-10.17]	4.59	[1.89-11.16]	6.30	[2.40-16.57]
Junior high school	1.42	[0.74-2.72]	0.96	[0.56-1.62]	1.59	[0.83-3.07]	0.54	[0.29-1.00]	1.42	[0.96-2.10]	2.89	[1.81-4.64]
High school	0.93	[0.49-1.78]	1.52	[1.01-2.29]	2.22	[1.33-3.73]	0.79	[0.46-1.35]	0.98	[0.68-1.41]	1.86	[1.18-2.93]
University	1		1		1		1		1		1	
Maternal occupation												
Farmer, craftswoman, merchant, entrepreneur	0.92	[0.25-3.39]	0.31	[0.08-1.14]	1.15	[0.26-5.04]	0.83	[0.22-3.14]	0.25	[0.08-0.75]	0.71	[0.17-2.86]
Management profession	0.97	[0.50-1.87]	0.61	[0.37-1.00]	1.12	[0.51-2.49]	0.64	[0.35-1.19]	0.67	[0.42-1.06]	0.47	[0.22-1.05]
Intermediate profession	1		1		1		1		1		1	
Manual worker	0.78	[0.27-2.23]	0.49	[0.21-1.14]	0.84	[0.34-2.05]	0.97	[0.46-2.04]	0.71	[0.41-1.20]	1.42	[0.83-2.45]
Unemployed	0.55	[0.18-1.76]	1.82	[0.84-3.93]	1.85	[0.75-4.57]	0.30	[0.08-1.08]	1.29	[0.70-2.39]	1.48	[0.76-2.88]
Missing	1.36	[0.74-2.51]	0.67	[0.40-1.13]	1.57	[0.92-2.69]	1.39	[0.84-2.30]	0.94	[0.67-1.33]	0.96	[0.62-1.50]
Smoking before/during pregnancy pregnancy)												
No smoking before or during	1		1		1		1		1		1	
Smoking before, but not during	0.92	[0.51-1.66]	0.91	[0.60-1.38]	0.77	[0.42-1.42]	0.99	[0.55-1.80]	0.47	[0.30-0.73]	0.61	[0.36-1.05]
Smoking before and during	1.24	[0.67-2.27]	0.50	[0.31-0.79]	0.56	[0.30-1.05]	1.27	[0.74-2.16]	0.99	[0.68-1.43]	0.65	[0.40-1.05]
Antenatal classes												
Attended	1		1		1		1		1		1	
Not attended	1.25	[0.70-2.22]	1.30	[0.83-2.03]	1.36	[0.80-2.31]	0.99	[0.65-4.52]	1.02	[0.76-1.38]	1.77	[1.16-2.72]

The model was also adjusted for gestational weight gain, gestational diabetes mellitus, hypertensive complications, delivery mode and infant’s birth weight (see Table 4)

**Table 4:** Association of maternal and neonatal outcome with prepregnancy BMI category (multinomial regression model stratified on parity) (*n* = 3149)

	Primiparous (*n* = 1335)						Multiparous (*n* = 1814)					
	Underweight		Overweight		Obesity		Underweight		Overweight		Obesity	
	*n* = 107		*n* = 214		*n* = 114		*n* = 130		*n* = 357		*n* = 197	
	OR	[95% CI]	OR	[95% CI]	OR	[95% CI]	OR	[95% CI]	OR	[95% CI]	OR	[95% CI]
Gestational weight gain												
Within IOM	1		1		1		1		1		1	
Below IOM	0.70	[0.42-1.16]	0.47	[0.26-0.85]	0.81	[0.44-1.50]	0.97	[0.63-1.51]	0.39	[0.26-0.60]	0.84	[0.53-1.32]
Above IOM	0.20	[0.10-0.38]	2.87	[1.93-4.26]	1.39	[0.82-2.37]	0.64	[0.37-1.10]	2.27	[1.68-3.06]	1.82	[1.20-2.76]
Gestational diabetes mellitus												
No	1		1		1		1		1		1	
Yes	0.27	[0.06-1.26]	1.51	[0.80-2.87]	2.80	[1.56-5.01]	0.27	[0.06-1.15]	3.43	[2.17-5.43]	5.16	[3.15-8.46]
Hypertensive complications												
No	1		1		1		1		1		1	
Yes	0.74	[0.16-3.37]	1.46	[0.62-3.43]	3.80	[1.83-7.89]	0.51	[0.06-4.14]	0.72	[0.28-1.88]	8.13	[3.97-16.64]
Delivery mode												
Vaginal	1		1		1		1		1		1	
Caesarean	0.77	[0.43-1.37]	1.38	[0.92-2.07]	1.33	[0.80-2.23]	0.39	[0.19-0.81]	1.28	[0.90-1.82]	1.80	[1.18-2.77]
Infant’s birth weight												
[2.5 kg-4 kg[	1		1		1		1		1		1	
≥ 4 kg	0.43	[0.06-2.94]	1.40	[0.70-2.77]	1.18	[0.36-3.86]	0.84	[0.38-1.87]	0.80	[0.52-1.22]	1.70	[1.03-2.80]
< 2.5 kg	2.47	[1.14-5.33]	1.03	[0.37-2.90]	0.83	[0.28-2.47]	4.93	[2.36-10.32]	0.48	[0.16-1.41]	0.56	[0.14-2.18]

The model was also adjusted for maternal age, maternal country of birth, education, occupation, smoking before and during pregnancy and antenatal class attendance (see Table 3)

### Sociodemographic factors associated with maternal obesity

Among the primiparous, women aged 25-29, when compared to those aged 30-34, were more likely to have obesity before pregnancy than to be of normal weight (*p*-value < 0.05). Those with a high school level were more likely to have obesity than to be of normal weight before pregnancy, compared to women with a university level.

Among the multiparous, women with a primary, junior high or high school level were more likely to have obesity than to be of normal weight before pregnancy compared to women with a university level (*p*-value < 0.05). Women who did not attend antenatal classes during pregnancy were more likely to have obesity before pregnancy than to be of normal weight.

### Maternal and fetal outcomes associated with maternal obesity

Among the primiparous, obesity before pregnancy, compared to a normal weight before pregnancy, was positively associated with GDM and hypertensive complications during pregnancy (*p*-value < 0.05). Among the multiparous, the odds of GDM, hypertensive complications, excessive GWG (vs GWG within IOM recommendations), caesarean section (vs. vaginal delivery) and infant birth weight ≥ 4 kg (vs. infant birth weight comprised between 2.5 and 4 kg) were higher for women with a pBMI ≥ 30.0 kg/m^2^ before pregnancy than for those with a pBMI comprised between 18.5 and 24.9 kg/m^2^ (*p*-value < 0.05).

As part of multinomial logistic regression, results for underweight and overweight women are also presented in Tables 3 (sociodemographic factors) and 4 (pregnancy outcomes).

### Sensitivity analyses

In the sample with non-imputed information concerning parity status, the same results were found (Additional file 2: Table S2 and Additional file 3: Table S3), excepting: (1) among the multiparous (*n* = 1648), the association was no longer statistically significant (*p*-value > 0.05) for birth weight ≥ 4 kg (OR = 1.67 [0.99-2.81], vs. 2.5 kg – 4.0 kg) (Additional file 3: Table S3); (2) among the primiparous (*n* = 1240), maternal age above 35 became significantly associated (*p*-value < 0.05) with prepregnancy obesity (OR = 2.27 [1.03-5.00], vs. 30-34 years) (Additional file 2: Table S2).

After removing covariates, infant birth weight, hypertensive complications and GDM, maternal obesity was still significantly associated (*p*-value < 0.05) with caesarian section (OR = 1.64 [1.09-2.46]) among multiparous women. Among primiparous women, maternal obesity was then significantly associated (*p*-value < 0.05) with caesarian section (OR = 1.69 [1.03-2.77]).

## Discussion

Our study identifies an extensive range of sociodemographic characteristics, health behaviors and maternal and neonatal outcomes independently associated with maternal obesity before pregnancy. Parity modulated the relationship between pBMI categories and numerous characteristics. Among primiparous women, maternal obesity was associated with a maternal age of 25-29 years, a high school education, and with GDM and hypertensive complications during pregnancy. Among the multiparous, maternal obesity was associated with primary, junior high and high school levels, and absence of attendance at antenatal classes. In this group, obesity before pregnancy was also associated with GDM, hypertensive complications, excess weight gain during pregnancy, caesarean section and infant birth weight ≥ 4 kg.

In our study, 10.2% of mothers had obesity before pregnancy. This prevalence is very close to the 9.9% prevalence observed in the French National Perinatal survey performed in 2010 [23]. The French National Perinatal survey was a nationwide survey that collected information on births after 22 weeks of amenorrhea, and also used self-reported prepregnancy weight and height several days after delivery [23]. However, the two studies, the French National Perinatal survey and the Epifane cohort, potentially underestimated the prevalence of obesity among French childbearing women. Indeed, several studies performed at the beginning of pregnancy [28, 29] showed that women who were overweight or with obesity before pregnancy tended to underestimate their weight. In addition, in Epifane, only dyads with a live infant born after 33 weeks were included. Since maternal obesity has been shown to be associated with greater risk of miscarriage [30] and premature delivery [31], obesity before pregnancy may have been underrepresented in Epifane. Compared to other studies performed in Western countries using self-reported anthropometric measures, the rate of obesity before pregnancy was close to that reported in Norway (8.8%) [32], but much lower than that reported in the US (24%) [33] or Australia (20%) [34].

For first-time mothers, we showed that a maternal age of 25-29 years compared to 30-34-year-old women was associated with obesity before pregnancy. A possible explanation would be that age at first birth might also reflect socioeconomic status. In Epifane, among primiparous women, 52.9% of women aged 25-29 had a university education, while this was the case for 65.5% of 30-34-year-old women. A positive association between maternal education and maternal age at first birth has been previously reported [35]. In addition, in Epifane, 12.3% of primiparous 25-29-year-old women held managerial positions, compared to 21.6% of 30-34–year-old primiparous women. We have adjusted analyses on such characteristics; however maternal education and occupation at birth might be insufficient to entirely capture the effect of socioeconomic status. Thus, unmeasured confounders and residual confounding cannot be ruled out, especially regarding women’s living conditions over life [36].

A low maternal education level was a risk factor of obesity before pregnancy, in accordance with previous studies [5, 37]. This result is also consistent with a study performed in the general French population, in which a low education level was associated with increased risk of obesity among adult women [38]. Women who are better educated and grow up under more favorable socioeconomic conditions may have had better nutritional knowledge and developed healthier behavior during child- and adulthood. Furthermore, mothers with a low education level may also be more likely to live in disadvantaged areas, shown in North America to be more obesogenic environments than affluent neighborhoods [39].

Parity also had an effect on the relationship between maternal obesity and attendance at antenatal classes. In Epifane, 76.2% of primiparous women and 35.9% of multiparous women attended antenatal classes. In France, seven antenatal classes are reimbursed by health insurance, in order to “contribute to the improvement of women, expectant mothers and newborn health” and to “encourage active involvement of the woman and the couple in their birth plan” [40]. Such classes provide comprehensive information on female physiology, conditions of delivery (position and gestures), and essential care during the infant’s first months (feeding, sleeping…), and proposed several approaches: obstetric psychoprophylaxis, yoga, aquatic gym, sophrology and so on. However, as in other countries [41, 42], participation in antenatal education has been shown to be closely related to socioeconomic status in France [43]. Single women [41], born in foreign countries [41, 42], with a low education level or occupational status [43] are less likely to participate in antenatal classes. However, after adjusting for sociodemographic factors, maternal obesity was still significantly associated with antenatal classes among multiparous women, suggesting another underlying mechanism. It was previously shown that obese women were more likely to have a negative perception of their bodies [44]. We may assume that obese multiparous women might have experienced uncomfortable feelings when attending such classes in a previous pregnancy, and subsequently decided to avoid them. Furthermore, we hypothesize that obese multiparous women may have suffered from complications such as the threat of premature delivery or fetal growth restrictions that were not addressed in our study, and felt these classes were less appropriate to their situation.

In studies performed among Danish [45] and American [46] women, mean weight gain during pregnancy was higher in primiparous than in multiparous women; in both strata of parity, women with higher BMI gained less on an average during their pregnancy than other pBMI groups. Due to the period during which these two cohorts were carried out (1996-2002 and 2005-2007, respectively), authors did not use 2009 IOM recommendations for GWG. These recommendations were updated in 2009, taking into account pBMI, and were aimed at decreasing the risk of post-partum weight retention, preterm birth, non-elective caesarean, GDM and pre-eclampsia [22]. In our study, primiparous gained more weight than multiparous (data not shown), and the average weight gain during pregnancy was the lowest in the highest pBMI categories. Nevertheless, although women with obesity before pregnancy gained less weight during their pregnancy than normal-weight women, they more often exceeded 2009 IOM recommendations, probably due to the lower threshold for meeting these guidelines when pBMI is high. We suggest that obese women who were less well educated confronted living conditions in which implementation of a healthy diet and regular physical activity is a challenge. Surprisingly, despite the same rate of obese women exceeding IOM recommendations among primiparous and multiparous women (47.5%), maternal obesity was associated with excessive GWG according to IOM recommendations only among multiparous women. This might be explained by the GWG in normal-weight women, which differs between primiparous and multiparous women. Indeed, 35.2% of normal-weight women exceeded IOM recommendations among primiparous women, while this was the case for 29.3% of normal weight women among the multiparous.

Maternal obesity before pregnancy was associated with infant birth weight ≥ 4 kg, but this association was statistically significant only among multiparous women. Both maternal obesity before pregnancy [4] and multiparity [3] are considered independent risk factors for infant birth weight above 4 kg. In the Epifane cohort, only newborns not transferred to another unit in the days following birth were included. A birth weight above 4,0-4,5 kg has been shown to be associated with increased risk of adverse perinatal issues, such as shoulder dystocia and perinatal asphyxia [47], requiring transfer to a neonatal intensive care unit. Thus, we probably underestimated the proportion of macrosomia in Epifane, as well as the association between maternal obesity and macrosomia.

Maternal obesity was also associated with caesarean delivery; however, this association was statistically significant only among multiparous women in the final model including infant birth weight, hypertensive complications and GDM. After removing these covariates in the sensitivity analyses, maternal obesity was also significantly associated with caesarean section among primiparous women. This suggests that they may be intermediate factors in the association of maternal obesity with caesarean section among primiparous women. We were unable to distinguish pre-labor caesarean section from emergency section, but a recent French study found an increased risk of pre-labor caesarean delivery for women with obesity only among the multiparous [48]. As those authors pointed out, this might be related to reluctance by the obstetrician to attempt vaginal delivery in women with obesity, in particular, multiparous women with previous caesarean section, for whom the rate of successful vaginal delivery is lower than for normal-weight women [49].

Consistent with other studies [3–5], obesity before pregnancy was associated with GDM and hypertensive complications in both primiparous and multiparous women. A meta-analysis performed in 2007 found that women with obesity had an unadjusted OR of 3.56 [3.05-4.21] for developing GDM compared to women of normal weight [50]. Many factors are involved in the relationship of maternal obesity with hypertensive complications and pre-eclampsia, such as insulin resistance, genetic factors, immunologic and infectious processes, but also lifestyle factors [51].

Limits and strengths of our study should be mentioned. First, based on the interaction test results, we stratified analyses on parity, thus sustaining its effect modification. However, this may have led to a decrease in statistical power. In order to limit the decrease in statistical power and bias selection due to non-random missing values, we imputed parity status. Sensitivity analyses without imputation showed only a few differences in results. Secondly, prepregnancy weight and height were collected at delivery, leading to an unmeasured level of recall bias. As previously mentioned, the fact that the mothers self-reported their prepregnancy weight and height may have led to misclassification, with risk of underestimating some associations. Nevertheless, it is difficult to correctly measure prepregnancy weight at a reasonable period before pregnancy. Weight at the first prenatal appointment (often occurring at the end of the first trimester of pregnancy in France) may reflect gestational weight gain at the beginning of pregnancy, which varies among women. The strength of our study lays in our sample analysis from a recent nation-wide birth cohort. In addition, we disposed of an extensive set of sociodemographic factors and high-quality data concerning outcomes, collected by midwives from health records.

## Conclusions

France has been confronted with an increase in prepregnancy body mass index since the seventies [52]; thus, identification of sociodemographic risk factors in maternal obesity is useful for implementing specific preventive action. Our study helps identify sociodemographic factors and health behavior related to prepregnancy obesity among primiparous and multiparous women. We have also highlighted an effect modification of parity in the association of prepregnancy obesity with maternal and neonatal outcomes, with higher risk in multiparous women. It has been shown that increased body mass index between the first and second pregnancy is associated with higher risk of maternal issues during the second pregnancy [53] and higher risk of stillbirth and infant mortality for the second newborn [54]. Our study design did not enable us to assess weight gain between the first and second pregnancy; longitudinal cohorts performed during a longer period will be useful for assessing changes in body mass index between the first and subsequent pregnancies. Identifying sociodemographic subgroups at risk of maternal obesity will enable targeting intervention aimed at reducing prepregnancy weight and maintaining healthy BMI between pregnancies. Maternal obesity is linked not only to lifestyle habits such as dietary intake and physical activity, but also to the social and physical environment. Development of effective actions, along with organization of pre- and postnatal care for primiparous and multiparous women with obesity, must take these factors into consideration.

## Additional files

Additional file 1: **Table S1.** Interactions between parity and covariates in their association with maternal pre-pregnancy BMI. (DOCX 15 kb)

Additional file 2: **Table S2.** Sensitivity analyses estimating the association of sociodemographic factors with prepregnancy BMI category in the sample with non-imputed information concerning parity status: multinomial regression model stratified on parity (*n* = 2888). (DOCX 25 kb)

Additional file 3: **Table S3.** Sensitivity analyses estimating the association of maternal and neonatal outcome with prepregnancy BMI category in the sample with non-imputed information concerning parity status: multinomial regression model stratified on parity (*n* = 2888). (DOCX 21 kb)
