# Supplementary material for: Sociodemographic factors and pregnancy outcomes associated with prepregnancy obesity: effect modification of parity in the nationwide Epifane birth-cohort
Source: BMC Pregnancy Childbirth. 2017 Aug 25;17:273. doi: 10.1186/s12884-017-1456-8 (PMC5574108; doi:10.1186/s12884-017-1456-8)
Supplement: Supplementary file 1 — Table S1. Interactions between parity and covariates in their association with maternal pre-pregnancy BMI. (DOCX 15 kb) [file 12884_2017_1456_MOESM1_ESM.docx]

Additional file 1: Table S1

|  | **Outcome : maternal pre-pregnancy BMI** |
| --- | --- |
|  | **n=3,149** |
|  | **p-value*** |
| Maternal age | 0.02 |
| Maternal education | 0.08 |
| Maternal country of birth | 0.33 |
| Maternal occupation | 0.63 |
| Smoking status before and during pregnancy | 0.08 |
| Antenatal classes attendance | 0.20 |
| Gestational Weight Gain | 0.26 |
| Gestational Diabetes mellitus | 0.12 |
| Hypertensive complications | 0.13 |
| Mode of delivery | 0.44 |
| Infant’s birth weight | 0.50 |

* p-value of interaction terms between parity (primiparous women/ multiparous women) and covariates (sociodemographic factors, health behaviors, and maternal and neonatal outcomes), in their association with maternal pre-pregnancy BMI in the adjusted multinomial logistic regression model.
