# Supplementary material for: Sociodemographic factors and pregnancy outcomes associated with prepregnancy obesity: effect modification of parity in the nationwide Epifane birth-cohort
Source: BMC Pregnancy Childbirth. 2017 Aug 25;17:273. doi: 10.1186/s12884-017-1456-8 (PMC5574108; doi:10.1186/s12884-017-1456-8)
Supplement: Supplementary file 2 — Table S2. Sensitivity analyses estimating the association of sociodemographic factors with prepregnancy BMI category in the sample with non-imputed information concerning parity status: multinomial regression model stratified on parity (n = 2888). (DOCX 25 kb) [file 12884_2017_1456_MOESM2_ESM.docx]

Additional file 2: Table S2

|  | Primiparous (n=1,240) | | | | | | | | | Multiparous (n=1,648) | | | | | | | | |
| --- | --- | --- | --- | --- | --- | --- | --- | --- | --- | --- | --- | --- | --- | --- | --- | --- | --- | --- |
|  | Underweight  (n=95) | | | Overweight  (n=203) | | | Obese  (n=104) | | | Underweight  (n=110) | | | Overweight  (n=326) | | | Obesity  (n=182) | | |
|  | OR | [95% | CI] | OR | [95% | CI] | OR | [95% | CI] | OR | [95% | CI] | OR | [95% | CI] | OR | [95% | CI] |
| Maternal age (years) |  |  |  |  |  |  |  |  |  |  |  |  |  |  |  |  |  |  |
| 18-24 | 1.98 | [0.90- | 4.39] | 0.85 | [0.50- | 1.43] | 1.52 | [0.65- | 3.53] | 3.62 | [1.75- | 7.49] | 0.75 | [0.36- | 1.55] | 1.12 | [0.54- | 2.31] |
| 25-29 | 1.47 | [0.80- | 2.71] | 0.67 | [0.44- | 1.02] | 2.17 | [1.13- | 4.18] | 1.46 | [0.89- | 2.41] | 1.64 | [1.17- | 2.30] | 1.21 | [0.77- | 1.90] |
| 30-34 | 1.00 |  |  | 1.00 |  |  | 1.00 |  |  | 1.00 |  |  | 1.00 |  |  | 1.00 |  |  |
| ≥35 | 0.86 | [0.31- | 2.40] | 0.71 | [0.37- | 1.37] | 2.27 | [1.03- | 5.00] | 0.79 | [0.43- | 1.45] | 1.07 | [0.74- | 1.53] | 1.50 | [0.95- | 2.37] |
| Maternal country of birth |  |  |  |  |  |  |  |  |  |  |  |  |  |  |  |  |  |  |
| France (mainland and overseas) | 1.00 |  |  | 1.00 |  |  | 1.00 |  |  | 1.00 |  |  | 1.00 |  |  | 1.00 |  |  |
| Maghreb and Sub-Saharan Africa | 1.04 | [0.31- | 3.52] | 1.31 | [0.50- | 3.45] | 1.10 | [0.35- | 3.52] | 0.57 | [0.18- | 1.77] | 1.26 | [0.74- | 2.12] | 0.68 | [0.38- | 1.23] |
| Europa, Asia, America, Oceania | 3.17 | [1.53- | 6.56] | 0.74 | [0.33- | 1.65] | 0.76 | [0.21- | 2.76] | 0.86 | [0.31- | 2.34] | 0.48 | [0.21- | 1.08] | 1.60 | [0.75- | 3.38] |
| Maternal education |  |  |  |  |  |  |  |  |  |  |  |  |  |  |  |  |  |  |
| Primary school | 1.53 | [0.23- | 10.15] | 2.14 | [0.62- | 7.45] | 1.06 | [0.14- | 8.03] | 6.06 | [1.61- | 22.78] | 4.67 | [1.60- | 13.63] | 6.68 | [2.15- | 20.72] |
| Junior high school | 1.37 | [0.69- | 2.73] | 0.82 | [0.47- | 1.44] | 1.58 | [0.83- | 3.00] | 0.63 | [0.32- | 1.23] | 1.22 | [0.80- | 1.84] | 2.68 | [1.65- | 4.36] |
| High school | 0.90 | [0.46- | 1.77] | 1.61 | [1.04- | 2.48] | 2.68 | [1.56- | 4.63] | 0.80 | [0.45- | 1.44] | 0.98 | [0.67- | 1.42] | 1.85 | [1.17- | 2.95] |
| University | 1.00 |  |  | 1.00 |  |  | 1.00 |  |  | 1.00 |  |  | 1.00 |  |  | 1.00 |  |  |
| Maternal occupation |  |  |  |  |  |  |  |  |  |  |  |  |  |  |  |  |  |  |
| Farmer, craftsman, merchant, entrepreneur | 1.03 | [0.28- | 3.82] | 0.32 | [0.09- | 1.19] | 1.25 | [0.29- | 5.45] | 0.81 | [0.20- | 3.26] | 0.25 | [0.08- | 0.78] | 0.75 | [0.19- | 3.00] |
| Management profession | 0.94 | [0.49- | 1.82] | 0.61 | [0.36- | 1.01] | 1.19 | [0.53- | 2.68] | 0.71 | [0.38- | 1.33] | 0.67 | [0.43- | 1.06] | 0.46 | [0.20- | 1.02] |
| Intermediate profession | 1.00 |  |  | 1.00 |  |  | 1.00 |  |  | 1.00 |  |  | 1.00 |  |  | 1.00 |  |  |
| Manual workers | 0.80 | [0.28- | 2.24] | 0.43 | [0.17- | 1.08] | 0.80 | [0.32- | 1.99] | 0.92 | [0.43- | 1.93] | 0.72 | [0.43- | 1.23] | 1.48 | [0.85- | 2.58] |
| Unemployed | 0.56 | [0.17- | 1.82] | 2.09 | [0.98- | 4.47] | 2.11 | [0.85- | 5.21] | 0.24 | [0.06- | 0.89] | 1.27 | [0.67- | 2.39] | 1.67 | [0.85- | 3.26] |
| Missing | 1.62 | [0.81- | 3.26] | 0.64 | [0.34- | 1.21] | 1.57 | [0.84- | 2.94] | 1.34 | [0.73- | 2.44] | 1.13 | [0.74- | 1.72] | 1.30 | [0.77- | 2.19] |
| Smoke before/ in pregnancy |  |  |  |  |  |  |  |  |  |  |  |  |  |  |  |  |  |  |
| No smoking before and during | 1.00 |  |  | 1.00 |  |  | 1.00 |  |  | 1.00 |  |  | 1.00 |  |  | 1.00 |  |  |
| Smoking before but not during | 0.89 | [0.48- | 1.66] | 0.85 | [0.56- | 1.30] | 0.82 | [0.44- | 1.55] | 0.96 | [0.50- | 1.85] | 0.52 | [0.33- | 0.82] | 0.70 | [0.41- | 1.20] |
| Smoking before and during | 1.24 | [0.63- | 2.42] | 0.46 | [0.28- | 0.76] | 0.58 | [0.30- | 1.10] | 1.27 | [0.72- | 2.26] | 0.92 | [0.61- | 1.38] | 0.59 | [0.34- | 1.00] |
| Antenatal classes |  |  |  |  |  |  |  |  |  |  |  |  |  |  |  |  |  |  |
| Attended | 1.00 |  |  | 1.00 |  |  | 1.00 |  |  | 1.00 |  |  | 1.00 |  |  | 1.00 |  |  |
| Not attended | 1.39 | [0.76- | 2.53] | 1.19 | [0.74- | 1.92] | 1.04 | [0.58- | 1.87] | 1.02 | [0.65- | 1.62] | 1.09 | [0.80- | 1.49] | 1.68 | [1.08- | 2.61] |

The model was also adjusted for gestational weight gain, gestational diabetes mellitus, hypertensive complications, delivery mode and infant’s birth weight
