# Supplementary material for: Sociodemographic factors and pregnancy outcomes associated with prepregnancy obesity: effect modification of parity in the nationwide Epifane birth-cohort
Source: BMC Pregnancy Childbirth. 2017 Aug 25;17:273. doi: 10.1186/s12884-017-1456-8 (PMC5574108; doi:10.1186/s12884-017-1456-8)
Supplement: Supplementary file 3 — Table S3. Sensitivity analyses estimating the association of maternal and neonatal outcome with prepregnancy BMI category in the sample with non-imputed information concerning parity status: multinomial regression model stratified on parity (n = 2888). (DOCX 21 kb) [file 12884_2017_1456_MOESM3_ESM.docx]

**Additional file 3: Table S3**

|  | Primiparous(n=1,240) | | | | | | | | | | | | | | | | | | | | | | Multiparous (n=1,648) | | | | | | | | | | | | | | | | | | | | | | | | | | |
| --- | --- | --- | --- | --- | --- | --- | --- | --- | --- | --- | --- | --- | --- | --- | --- | --- | --- | --- | --- | --- | --- | --- | --- | --- | --- | --- | --- | --- | --- | --- | --- | --- | --- | --- | --- | --- | --- | --- | --- | --- | --- | --- | --- | --- | --- | --- | --- | --- | --- |
|  | Underweight  (n=95) | | | | | Overweight  (n=203) | | | | | | | | Obesity  (n=104) | | | | | | | | | Underweight  (n=110) | | | | | | | | | Overweight  (n=326) | | | | | | | | | Obesity  (n=182) | | | | | | | | |
|  | OR | [95% CI] | | | | OR | | | [95% CI] | | | | | OR | | | [95% CI] | | | | | | OR | | | [95% CI] | | | | | | OR | | | [95% CI] | | | | | | OR | | | [95% CI] | | | | | |
| Gestational weight gain |  |  | |  | |  | | |  | | |  | |  | | |  | | |  | | |  | | |  | | |  | | |  | | |  | | |  | | |  | | |  | | |  | | |
| Within IOM | 1.00 |  | |  | | 1.00 | | |  | | |  | | 1.00 | | |  | | |  | | | 1.00 | | |  | | |  | | | 1.00 | | |  | | |  | | | 1.00 | | |  | | |  | | |
| Below IOM | 0.89 | [0.53- | | 1.49] | | 0.39 | | | [0.21- | | | 0.71] | | 0.70 | | | [0.37- | | | 1.33] | | | 0.87 | | | [0.55- | | | 1.39] | | | 0.36 | | | [0.23- | | | 0.56] | | | 0.75 | | | [0.47- | | | 1.20] | | |
| Above IOM | 0.25 | [0.13- | | 0.50] | | 2.92 | | | [1.94- | | | 4.39] | | 1.44 | | | [0.82- | | | 2.52] | | | 0.63 | | | [0.35- | | | 1.15] | | | 2.14 | | | [1.57- | | | 2.92] | | | 1.93 | | | [1.26- | | | 2.95] | | |
| Gestational diabetes mellitus |  |  | |  | |  | | |  | | |  | |  | | |  | | |  | | |  | | |  | | |  | | |  | | |  | | |  | | |  | | |  | | |  | | |
| No | 1.00 |  | |  | | 1.00 | | |  | | |  | | 1.00 | | |  | | |  | | | 1.00 | | |  | | |  | | | 1.00 | | |  | | |  | | | 1.00 | | |  | | |  | | |
| Yes | 0.26 | [0.06- | | 1.23] | | 1.68 | | | [0.88- | | | 3.23] | | 3.13 | | | [1.73- | | | 5.66] | | | 0.30 | | | [0.07- | | | 1.31] | | | 3.17 | | | [1.96- | | | 5.11] | | | 4.94 | | | [2.95- | | | 8.28] | | |
| Hypertensive complications |  |  | |  |  | | |  | | |  | |  | | |  | | |  | | |  | | |  | | |  | | |  | | |  | | |  | | |  | | |  | | |  | | |  |
| No | 1.00 |  | |  | | 1.00 | | |  | | |  | | 1.00 | | |  | | |  | | | 1.00 | | |  | | |  | | | 1.00 | | |  | | |  | | | 1.00 | | |  | | |  | | |
| Yes | 0.50 | [0.08- | | 3.28] | | 1.65 | | | [0.66- | | | 4.14] | | 4.80 | | | [2.25- | | | 10.23] | | | 0.58 | | | [0.07- | | | 5.05] | | | 0.76 | | | [0.27- | | | 2.11] | | | 10.07 | | | [4.77- | | | 21.24] | | |
| Delivery mode |  | |  | | |  |  | | |  | | | |  |  | | |  | | |  | | |  | | |  | | |  | | |  | | |  | | |  | | |  | | |  | | |  |  |
| Vaginal | 1.00 |  | |  | | 1.00 | | |  | | |  | | 1.00 | | |  | | |  | | | 1.00 | | |  | | |  | | | 1.00 | | |  | | |  | | | 1.00 | | |  | | |  | | |
| Caesarian | 0.71 | [0.39- | | 1.31] | | 1.47 | | | [0.97- | | | 2.24] | | 1.50 | | | [0.88- | | | 2.56] | | | 0.51 | | | [0.25- | | | 1.06] | | | 1.16 | | | [0.80- | | | 1.70] | | | 1.80 | | | [1.16- | | | 2.82] | | |
| Infant’s birth weight |  |  | |  | |  | | | |  | | | |  |  | | |  | | |  | | |  | | |  | | |  | | |  | | |  | | |  | | |  | | |  | | | |  |
| [2.5kg-4kg[ | 1.00 |  | |  | | 1.00 | | |  | | |  | | 1.00 | | |  | | |  | | | 1.00 | | |  | | |  | | | 1.00 | | |  | | |  | | | 1.00 | | |  | | |  | | |
| ≥4 kg | 0.46 | [0.07- | | 3.15] | | 1.23 | | | [0.59- | | | 2.56] | | 1.19 | | | [0.36- | | | 3.94] | | | 0.75 | | | [0.33- | | | 1.75] | | | 0.84 | | | [0.54- | | | 1.30] | | | 1.67 | | | [0.99- | | | 2.81] | | |
| <2.5kg | 2.50 | [1.16- | | 5.38] | | 1.11 | | | [0.37- | | | 3.31] | | 0.61 | | | [0.18- | | | 2.01] | | | 5.07 | | | [2.33- | | | 11.00] | | | 0.41 | | | [0.12- | | | 1.42] | | | 0.62 | | | [0.19- | | | 2.01] | | |

Model was also adjusted for maternal age, maternal country of birth, education, occupation, smoking before and during pregnancy and antenatal class
